# Project ACHIEVE – using implementation research to guide the evaluation of transitional care effectiveness

**DOI:** 10.1186/s12913-016-1312-y

**Published:** 2016-02-19

**Authors:** Jing Li, Jane Brock, Brian Jack, Brian Mittman, Mary Naylor, Joanna Sorra, Glen Mays, Mark V. Williams, for the Project ACHIEVE Team

**Affiliations:** Administrative Director of the Center for Health Services Research, Assistant Professor of Internal Medicine, University of Kentucky, Lexington, KY USA; Care Transitions Theme Support Center, Telligen, Englewood, CO USA; Family Medicine, Boston University School of Medicine, Boston, MA USA; Research Scientist, Research and Evaluation, Kaiser Permanente, Pasadena, CA USA; US Department of Veterans Affairs Greater Los Angeles Healthcare System, VA Center for Implementation Practice and Research, Los Angeles, CA USA; UCLA School of Medicine, UCLA Clinical Translational Science Institute, Los Angeles, CA USA; Director of NewCourtland Center for Transitions and Health, University of Pennsylvania School of Nursing, Philadelphia, PA USA; Westat, Washington, D.C. USA; National Coordinating Center for Public Health Services & Systems Research, University of Kentucky, Lexington, KY USA; Center for Health Services Research, Department of Internal Medicine, University of Kentucky, Kentucky Clinic J525, Lexington, KY 40536-0284 USA

**Keywords:** Transitional care, Implementation research, Comparative effectiveness, Patient centered

## Abstract

**Background:**

Poorly managed hospital discharges and care transitions between health care facilities can cause poor outcomes for both patients and their caregivers. Unfortunately, the usual approach to health care delivery does not support continuity and coordination across the settings of hospital, doctors’ offices, home or nursing homes. Though complex efforts with multiple components can improve patient outcomes and reduce 30-day readmissions, research has not identified which components are necessary. Also we do not know how delivery of core components may need to be adjusted based on patient, caregiver, setting or characteristics of the community, or how system redesign can be accelerated.

**Methods/design:**

Project ACHIEVE focuses on diverse Medicare populations such as individuals with multiple chronic diseases, patients with low health literacy/numeracy and limited English proficiency, racial and ethnic minority groups, low-income groups, residents of rural areas, and individuals with disabilities. During the first phase, we will use focus groups to identify the transitional care outcomes and components that matter most to patients and caregivers to inform development and validation of assessment instruments. During the second phase, we will evaluate the comparative effectiveness of multi-component care transitions programs occurring across the U.S. Using a mixed-methods approach for this evaluation, we will study historical (retrospective) and current and future (prospective) groups of patients, caregivers and providers using site visits, surveys, and clinical and claims data. In this natural experiment observational study, we use a fractional factorial study design to specify comparators and estimate the individual and combined effects of key transitional care components.

**Discussion:**

Our study will determine which evidence-based transitional care components and/or clusters most effectively produce patient and caregiver desired outcomes overall and among diverse patient and caregiver populations in different healthcare settings. Using the results, we will develop concrete, actionable recommendations regarding how best to implement these strategies. Finally, this work will provide tools for hospitals, community-based organizations, patients, caregivers, clinicians and other stakeholders to help them make informed decisions about which strategies are most effective and how best to implement them in their communities.

**Trial registration:**

Registered as NCT02354482 on clinicaltrials.gov on 1/29/2015

## Background

Though improving transitional care (TC) is a patient safety and quality improvement priority with numerous national initiatives implementing various combinations of interventions to improve the quality of care transitions [1–6], patients and caregivers still encounter fragmented and disorganized care when moving between health care settings. Poorly executed, non-standardized transitions result in a multitude of adverse effects with wide ranging consequences for both patients and their caregivers [7, 8]. Problematic transitions occur from and to virtually every type of health care setting, but especially when patients leave the hospital to receive care in the post-acute setting (e.g., independent rehabilitation or skilled nursing facilities - SNFs) or at home. Systems issues need to be addressed, including: communication of unresolved problems among providers, patient education regarding medications and treatments, monitoring medication reconciliation and adherence, arranging appropriate follow-up and monitoring the status of patients soon after discharge including adverse drug events [9].

The hospital discharge is a complex, multi-step process requiring integrated communications among the inpatient care team, the patient and caregiver, the outpatient care team, and community services [10]. Research has shown for many years that when managed poorly, this process yields lowered patient and caregiver satisfaction, adverse events, unnecessarily high utilization of subsequent health services, increased rates of potentially avoidable hospitalizations, and other gaps in quality and safety [7, 8, 11–13]. While some readmissions are unavoidable or unanticipated, such as those resulting from the inevitable progression of disease or worsening of chronic conditions [14], readmissions also result from poor quality of care or inadequate communication between care settings [15].

Lack of assessment of patient and contextual issues represents an important gap in evidence regarding TC programs. While researchers have worked to develop interventions that can improve care transitions, few have looked to the promise of enhanced transitional care with an emphasis on what is most meaningful and important to patients and family caregivers [16].

Moreover, efforts at coordination across the continuum of care face difficult barriers, especially given the unique aspects of each patient (e.g. socioeconomic status, caregiver support, health literacy), and given that specific factors contributing to ineffective care transitions often differ among health and community-based organizations and contexts. To apply findings from the current widespread experimentation to improve transitional care requires a comprehensive evaluation using comparative effectiveness research techniques. In response to the Patient Centered Outcome Research Institute (PCORI) Transitional Care funding announcement, we proposed and received the award for Project ACHIEVE (Achieving Patient-Centered Care and Optimized Health In Care Transitions by Evaluating the Value of Evidence). This project aims to learn from patients and caregivers which transitional services and outcomes matter most to them, rigorously evaluate current efforts at improving care transitions, and develop recommendations on best practices for patient-centered care transition interventions and guidance for scalability and large-scale dissemination.

## Study design

Following NIH recommendations on mixed-methods research [17], Project ACHIEVE is undertaking qualitative and quantitative studies that build upon and complement each other including: (1) qualitative research to identify the transitional care components (TCCs) and outcomes that matter most to patients and their caregivers; (2) qualitative research to identify healthcare provider and community contextual factors that influence implementation of TCCs; (3) retrospective secondary data analyses (longitudinal comparative) of patients experiencing TCCs based on exposure through the national programs [e.g., Partnership for Patients’ Hospital Engagement Networks (HENs), the Quality Improvement Organizations’ Integrating Care for Populations and Communities (QIO ICPC) Aim and the Centers for Medicare & Medicaid Innovation’s Community-based Care Transitions Programs (CCTPs)]; and (4) using mixed methods to conduct a prospective cohort analysis of patients and caregivers exposed to defined clusters of TCCs to determine which are most associated with the outcomes that matter most to patients and caregivers. The proposed approach is a natural experiment observational study. With consistent, proactive input from patients, caregivers and stakeholders, the proposed study will rigorously evaluate current TC efforts and develop recommendations on best practices for patient-centered care transitions and guidance for implementation and scalability. Project ACHIEVE’s long-term objective will be to provide detailed, evidence-based, comprehensive guidance for the design, appropriate adaptation, implementation and sustainment of TC programs for improving patient and caregiver outcomes in diverse healthcare and community contexts.

### Ethics and consent

Project ACHIEVE was approved by the Institutional Review Board (IRB) at the University of Kentucky (IRB Number 14–0789-F3R). Each partner site also obtained approval from its own institution’s IRB for its study activities such as focus groups or survey administration. Informed consent will be obtained from all focus group participants. Patients enrolled in the survey will provide HIPAA authorization for release of their personal health information to enable collaborators on the ACHIEVE team to contact them for recorded verbal consent and survey conduct.

### Patient population

Project ACHIEVE will focus on Medicare fee-for-services beneficiaries and study diverse high risk patient populations, including those with: 1) multiple chronic conditions; 2) mental health issues; 3) rural area domicile; 4) limited English proficiency or low health literacy; 5) low socioeconomic status; 6) Medicare and Medicaid dual eligible; and 7) disabled and younger than 65. Project ACHIEVE will primarily focus on patient transitions from hospital to home, but will also evaluate transitions to and from skilled nursing facilities and home with a focus on Medicare beneficiaries. This expanded evaluation is necessary given the proportion (20 %) of older adults who move from hospital to SNFs and then to long term care or home [18, 19].

### Project ACHIEVE framework

**Fig. 1:**
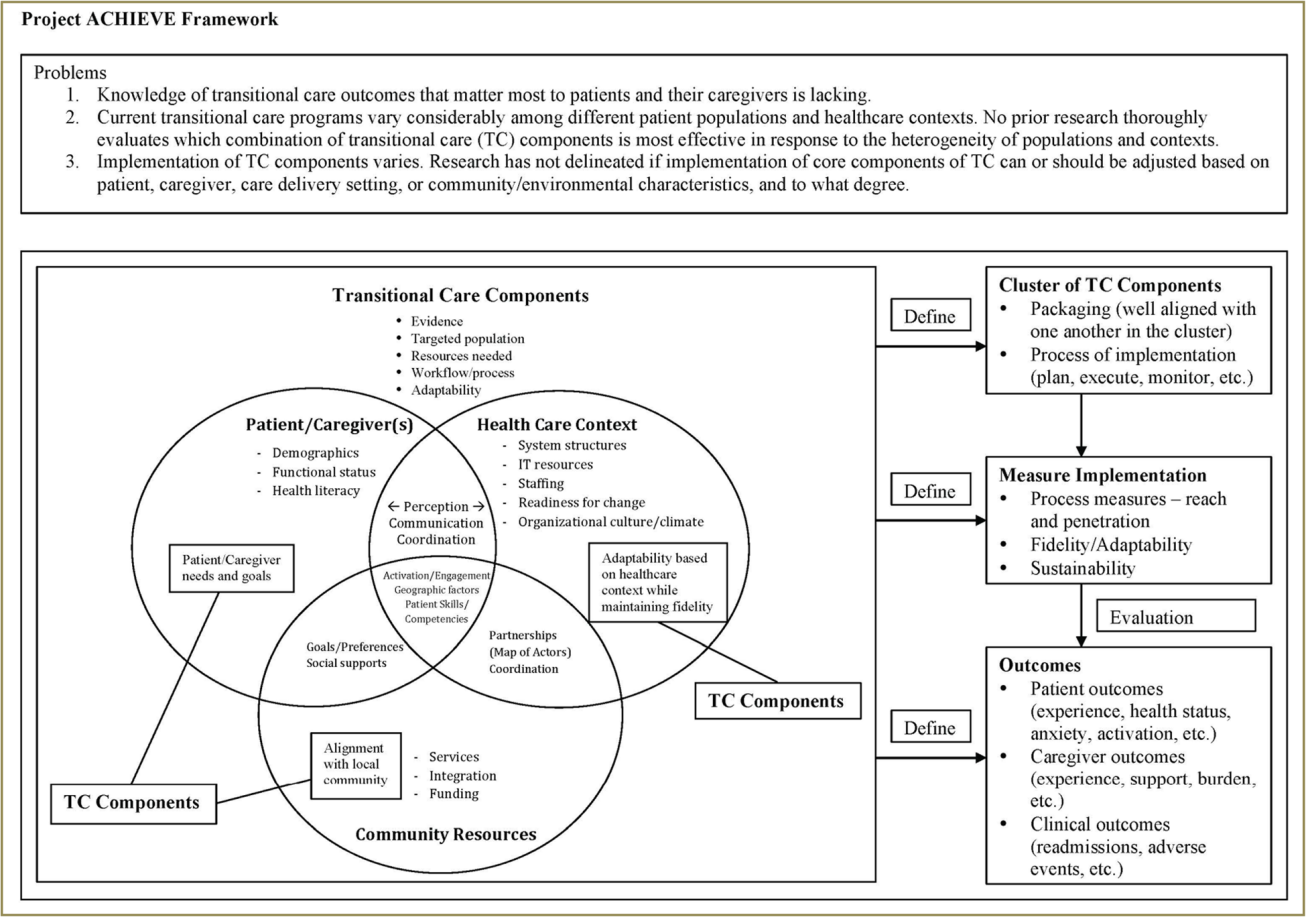
Project ACHIEVE Framework – Based on the Consolidated Framework for Implementation Research (CFIR)

The research team reviewed several previously developed conceptual frameworks on diffusion and dissemination of evidence-based practices including the promoting action on research implementation in health services (PRAiHS) framework [20], interactive systems framework (ISF) [21], consolidated framework for implementation research (CFIR) [22], and RE-AIM framework [23]. PRAiHS focuses on successful implementation as a function of evidence, context, and facilitation. ISF highlights the roles of key actors in the dissemination process. The RE-AIM framework, though developed for evaluation, is widely used to provide organizing principles for the dissemination of evidence-based practices. CFIR is a recently developed framework that synthesizes several existing frameworks and focuses on constructs related specifically to implementation and subsequent routinization. Building on existing evidence in transitional care and ongoing work of Project ACHIEVE’s research team, we developed a Project ACHIEVE framework (Fig. 1) based on CFIR. This Project ACHIEVE framework outlines our structured, phased approach to determine which transitional care service clusters are most effective in improving patient-centered outcomes in different at-risk subpopulations and in different healthcare contexts. The framework in Fig. 1 shows four major domains (TCCs, patients/caregiver(s), health care context, and community resources), and conveys how these domains interact in rich and complex ways to influence patient outcomes.

### Specific aims

Below we delineate the specific methodology and study design for each of Project ACHIVE’s specific aims.

#### Specific Aim 1– Identify the transitional care outcomes and components that matter most to patients and caregivers

**Fig. 2:**
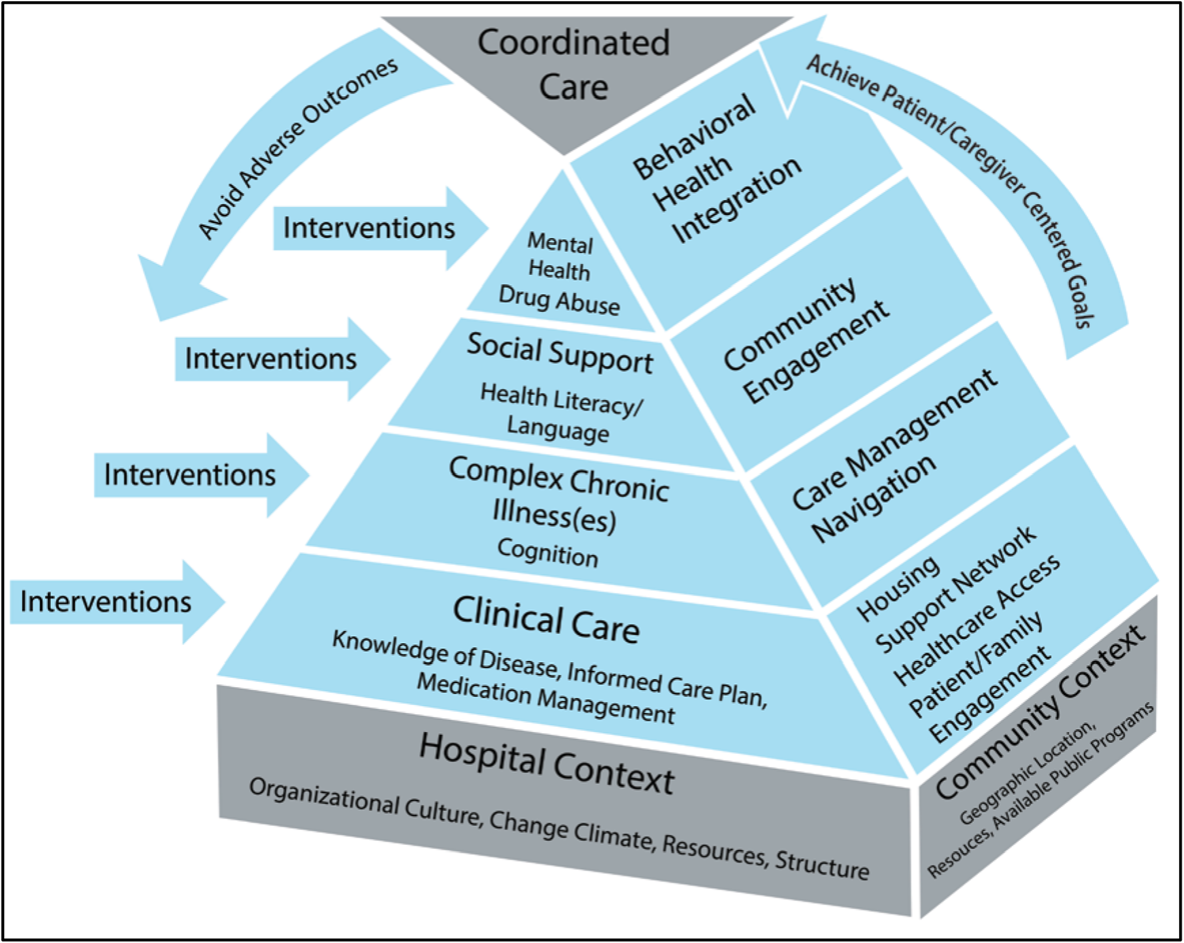
Project ACHIEVE Model for Coordinated Care

Project ACHIEVE’s Model for Coordinated Care (Fig. 2) identifies targets for interventions and important aspects of hospital and community contexts that influence effectiveness of interventions and their implementation. The preliminary list of TCCs we plan to evaluate is based on current available literature and input from experts in transitional care, and will be refined based on findings from focus groups and surveys of patients, caregivers and providers. Using a hospital TCC adoption survey and site visits, we will determine how TC improvement efforts were selected, what components were deployed and whether they were modified based on the local delivery system, the nature of the population served, and other contextual factors. We believe that to effectively and safely improve TC, it will be important to integrate a patient- and caregiver-centered approach in four transitional care intervention categories: 1) Patient and Caregiver Engagement/Education; 2) Transition Management with Information Exchange; 3) Clinical/Medication Issues; 4) Follow-up Care Throughout Episode of Acute Illness. We expect that clusters of TCCs would include components addressing each of these categories.

Much of the current available data influencing the readmission discourse has come from large administrative data sets. Additional highly relevant information can be gained by exploring patient and caregiver perspectives on care transitions through focus groups, garnering unique insights leading to more patient- and caregiver-centered interventions. Our proposed patient and caregiver focus groups will occur at multiple sites to ensure diversity including: Boston, Philadelphia, Colorado, Southern California, Kentucky, Louisiana and elsewhere as needed. To understand patients’ and caregivers’ experiences of care transitions and determine how well specific TCCs meet individual healthcare needs, we will use mixed methods with a community-based participatory research (CBPR) approach [24–26]. This includes qualitative analyses of focus groups and key informant interviews, and quantitative analyses of patient and caregiver outcomes that are collected through survey conduct and claims data.

##### Analysis

We will analyze qualitative data using the constant comparative method to determine patients’ perspectives on the care transition experience, and we will analyze quantitative data using frequency analysis to determine factors associated with both poor and optimal outcomes from the patients’ and caregivers’ perspective. NVIVO 10 software (QSR International Pty. Ltd., Melbourne, Australia) will be used for qualitative data management and analysis. We will employ the constant comparative method of qualitative data analysis [27]. A team of three investigators with expertise in care transitions, healthcare communication and qualitative research will independently analyze the transcripts, using grounded theory, to code data and identify themes. Discrepancies will be resolved through negotiation. We will develop codes iteratively, and refine them to identify conceptual segments of data. Initial codes will be analyzed and consolidated into dominant themes and a codebook will be developed. Recurring themes will be identified as patient/caregiver-centered care transition priorities and preferences for healthcare services during care transitions. The final results will be presented to our Stakeholder Advisory Group (SAG) for validation and feedback. The SAG consists of 25 individuals representing patients, caregivers, health care providers, advocacy organizations, professional associations, and payers/policymakers. Once finalized, the data will be used for development of the patient and caregiver survey for the prospective cohort analysis of patients and caregivers exposed to pre-defined clusters of TCCs.

#### Specific Aim 2 – Determine which evidence-based transitional care components (TCCs) or clusters most effectively yield patient and caregiver desired outcomes overall and among diverse patient and caregiver populations in different types of care settings and communities

The comparisons to be supported within the retrospective and prospective analyses are designed to identify the individual and combined effects of key TC components that are most highly valued by patients and that are included in formal TC models already in use today through national programs. A traditional clinical trials approach to this research design would seek to estimate TC component effects and interactions using a full factorial experiment; however, the large number of possible combinations of TC components that a patient may receive based on the clinical and community setting in which they receive care renders such an approach infeasible under the time and resource constraints of this study. As an alternative, we use a fractional factorial (FF) study design to specify comparators and estimate the individual and combined effects of key TC components. Fractional factorial designs have traditionally been used in designed experiments where the treatment groups are pre-specified. In our observational study, we will not necessarily have all the groups for a FF, but this design will allow us to know which higher order interactions are confounded with lower order effects, which will result in better interpretation of our results.

#### Retrospective longitudinal comparative analyses

This analysis exploits variation in the incidence and timing of TCC cluster adoption in order to examine which types of patients and hospitals achieve the greatest improvements under which TCCs and clusters.

##### Data sources

1) Medicare claims data; 2) Kaiser Permanente Southern California (KPSC) will provide clinical data from its EHR regarding process and outcome measures and claims data across KPSC medical centers involved in their TC efforts; 3) Health Research & Educational Trust (HRET), Essential Hospitals Institute (EHI), and Joint Commission Resources (JCR) are three HENs engaging about 1800 hospitals to reduce rehospitalizations, and they will provide data on what TCCs hospitals implemented, their readmission rates over time and hospital demographics.

##### Analysis

Our data sources allow for identification and examination of process measures to evaluate the success of implementation of TCCs and adaptation of established models, as well as measurement of pre-implementation/post-implementation changes in care delivery patterns and outcomes. We will conduct pooled analyses of all patients as well as focused analyses of ACHIEVE targeted patient populations. A retrospective longitudinal cohort design will be employed. Hospitals that implemented different clusters converted from pre status to post status at different times during the study period, enhancing the study’s ability to distinguish program effects from general temporal trends in patient care. Hospital level (e.g., Heckman selection-correction models) and patient level (e.g., propensity score) analytic strategies will be used to address the possibility of non-equivalence among hospitals and their patients. Fixed-effects, difference-in-difference estimation will be used to compare pre-post changes in outcomes between TCC cluster implementing and non-implementing hospitals while controlling for baseline differences in patient and hospital characteristics. Hierarchical general linear latent and mixed models (GLLAMM) will account for the clustering of patients within hospitals and the temporal correlation among observations. Marginal structural model specifications and instrumental variables analysis will be used to provide further protection against selection bias and confounding on unobserved patient heterogeneity, following PCORI’s methodological standards for patient-centered outcomes research [28]. These analyses will use instrumental variables that influence a patient’s probability of exposure to TCCs but that have no independent influence on network continuity and other outcome measures, except through the causal pathway.

#### Prospective analyses

Using information from the focus groups and retrospective analysis, we will undertake a prospective cohort analysis of patients and caregivers exposed to pre-defined clusters of TCCs.

##### Survey development

Based on results from focus groups, an updated review of available literature, input from Scientific and Stakeholder Advisory teams, and ACHIEVE’s preliminary findings, we will identify key domains/subdomains, items and measures to include in the development of patient, caregiver and provider surveys. In addition to demographic information, we anticipate that the surveys will include some of the following domains and measures: a) Patient—experience, involvement in care, engagement, receipt and understanding of care plan and medications, access to transportation and care, assessment of the various TCCs, extent to which patient needs were met, follow-up primary care provider visit, follow-up lab and tests, community services connection and usage, adverse drug events, 30-day emergency department visits and rehospitalizations; b) Caregiver—experience, involvement in care, anxiety, burden, understanding of patient’s care plan and medications, caregiver assessment of extent to which patient’s needs and their own needs were met, access to support; c) Provider—facilitators and barriers to implementing TCCs, provider assessment of various TCCs (perceived value for their institutions and patients, feasibility and compatibility with local resources, etc.), adaptation of TCCs, organizational contexts (leadership and physician engagement, change culture, etc.), community collaboration.

##### Hospital and community recruitment

Identification of appropriate and diverse hospitals and community-based organizations will be essential. Recognizing that hospital and community contexts do not remain static, we propose to develop a purposively selected hospital list for recruitment. We will select hospitals to ensure representation of: 1) urban and rural areas; 2) safety-net; 3) critical access; 4) integrated delivery system; 5) involvement in care delivery demonstrations (e.g., accountable care organization, bundled payments for care improvement). Participating hospitals will assist the ACHIEVE research team to identify and recruit eligible patients, caregivers, and providers to complete surveys and provide necessary clinical data through a secured mechanism.

##### Sampling strategy

Using a fractional factorial design, hospitals will serve as the primary sampling unit, with selection determining patients/caregivers available for further study. Hospitals will be selected so that a sufficient number/combination of TCC clusters can be compared (this provides connectedness for the fractional factorial design) for a subpopulation. Specifically, patients will be sampled from their respective hospital (stratified random samples by subpopulation) and outcomes compared based on TCC clusters received. Replication within a TCC cluster will allow for comparison of TCCs while accounting for the hospital-effect. Use of the fractional factorial relies heavily on having enough hospitals and TCCs for sufficient combinations to yield a connected design (estimable differences). Therefore, in the selection of hospitals, availability of subpopulations and presence of particular TCCs will be carefully considered.

##### Sample size calculations

To provide flexibility for the dynamic study design where outcomes and TCC clusters are identified in year 1, several conditions were considered, e.g. continuous outcomes are expected but binary outcomes are also possible. The base sample size was estimated using contrasts within a one-way ANOVA then augmented to account for subpopulation analyses and nesting of patients within hospitals. While comparisons will be made overall, of greater interest is the comparison of TCC clusters specific to particular subpopulations, and the overall sample size will need to be sufficiently large to allow for comparisons within these smaller contexts. Additionally, the recruitment of patients nested within hospitals may impact the independence of observations and sample size estimates should also be augmented by the design effect. To allow for sufficient heterogeneity in the sample, we assumed that we would need approximately 6 such TCC clusters. To account for the nesting of patients within hospitals while comparing multiple TCC clusters for particular subpopulations, 300 patients will be recruited in 40 hospitals for a total sample size of 12,000 patients.

##### Survey administration and data collection

For survey administration and data collection we will use computer-assisted telephone interviewing (CATI) to administer patient and caregiver surveys. After receiving a list of patients from participating hospitals, the research team will call patients and caregivers between 7 and 45 days post discharge to assess delivery of transitional care services and to ensure patient and caregiver recall about post-discharge care and events. To obtain high response rates, we will incorporate steps from the Dillman tailored design method [29] for administering telephone and web surveys. Our proposed protocol is to identify eligible patients while hospitalized and obtain HIPAA authorization to share their contact information with the research team. Then, we will mail pre-notification letters from collaborating hospitals to patients to inform them that they will be contacted to complete a survey about the care that was provided during hospitalization and post-discharge. Patients will be informed that the research team is also interested in surveying their family caregiver by phone (i.e., snowball sampling). Up to 14 call attempts will be made and if a patient is unable to answer questions by phone, a proxy who can assist the patient or respond on behalf of the patient will be sought. The total number of completed patient surveys will be 12,000, and we plan for up to 7,200 completed caregiver surveys.

##### Prospective claims and clinical data collection

Medicare Fee-for-Services claims data will be collected through re-use agreement with CMS or ResDAC. Participating hospitals will submit clinical data on a monthly basis through a secured web-based platform. And KPSC will provide data for its 10 medical centers through its system integrated electronic health record.

##### Survey data analysis

Survey data will be compiled and cleaned, and descriptive statistics will be generated from the survey results. Psychometric analyses will also be conducted to examine items for variability of response, calculate reliability statistics and item inter-correlations, and examine the factor structure of the measures. Composite scores will be created and the survey data will then be used to examine the study’s aims and hypotheses regarding TC delivery and outcomes.

##### General statistical considerations

Continuous variables will be summarized with descriptive statistics (n, mean, standard deviation, median, first and third quartiles, and min and max); categorical variables will be described with counts and percentages. Numerical and graphical summaries will be provided overall, by subpopulation context, and by TCC and cluster. Simple comparisons of groups will be made using ANOVA for continuous variables and chi-square tests for categorical outcomes. Given the hierarchical nature of the selection of patient/caregivers through hospitals, primary analyses will utilize hierarchical models to investigate the relationship of outcomes to both patient/caregiver and hospital characteristics; GEEs (generalized estimating equations) and GLMMs (general linear mixed model) will be the primary models used.

#### Specific Aim 3 – identify barriers and facilitators to the implementation of specific TCCs or clusters of TCCs for different types of care settings and communities

##### Provider focus groups

The provider interview guide is based on determination of key contextual factors influencing TCC intervention selection and adaptation. Examples of likely contextual factors include: performance of a root cause analysis before selecting interventions; rural vs. urban location; poverty concentration in the served area; presence of and trust in partners across the care continuum; availability of electronic information exchange infrastructure; use of common improvement tracking measures and/or common risk identifiers; establishment of cross-continuum case review committees; etc. We will perform group interviews with 20 groups of 3 to 5 people from existing community programs, selecting communities that vary by geographic location, size, urbanicity/rurality and poverty concentration. These could be 3 to 5 people from a single provider (in the case of hospital led and delivered interventions) or from a group of providers (representatives from cross-continuum teams in the case of multi-provider or cross-setting interventions), to test the validity of identified contextual features.

##### Provider survey

The survey will be conducted through web mode and mailings to collect information on barriers and facilitators in different organizations and evaluate the quality of transfer processes and transitional care where applicable. Both hospital-based (e.g., hospitalists) and post-acute or community providers will be targeted. We will use Medicare claims data to identify the providers who next see patients discharged from any given hospital, generate diagrams of referral patterns using UCINET software, and count referrals per provider.

##### Site visits

The purpose of hospital site visits will be to validate hospital self-reporting, assess the implementation of TCCs, seek feedback on study protocol and process, evaluate hospital contextual factors impact such as leadership commitment, teamwork structure, physician engagement, staffing, etc., and identify barriers and facilitators. Site selection strategy is to include a representative mix of candidates based on geographic regions, population setting, ownership characteristics of organization, community or health system size, population served, and transitional care models/programs implemented. During the visit, our research team will meet with different teams to conduct semi-structured interviews. The teams include leadership and management, transitional care implementation team, internal stakeholders and partners, and post-acute and community partners.

#### Specific Aim 4 Develop recommendations for dissemination of the research findings on the best evidence regarding how to achieve optimal TC services and outcomes to patients, caregivers and providers

Based on study findings (with guidance from patients, caregivers, and our partners), we plan to develop a toolkit to guide hospitals, post-acute providers and community organizations on implementing TCC clusters effectively, aligned with the characteristics of patient populations they serve and local health and community contexts. The ACHIEVE team will also develop multiple whitepapers to provide resources to patients and caregivers and engage them in the TC process.

The members of ACHIEVE team have access to multiple venues for knowledge and experience dissemination. In the last year of the study, we will finalize a large-scale dissemination and implementation plan. The ACHIEVE team will share the study findings (focus groups, retrospective data analysis, surveys, and prospective analysis) through webinars, seminars, workshops, and presentations at national conferences when final results are available. We will also prepare manuscripts for peer-reviewed publications to include a description of the project’s methodology; the findings from focus groups, surveys, effectiveness of TC clusters on outcomes; barriers and facilitators of TCC implementation; and description of the best clusters of TCCs for different patient subpopulations.

## Discussion

Traditionally, efforts to reduce readmissions have focused on hospitals, but experts now recognize that multiple factors along the care continuum influence readmissions and must be addressed in a comprehensive manner [30]. Capitalizing on the opportunity for a natural experiment observational study using rigorous qualitative and quantitative methods (secondary data analyses and by directly surveying patients, caregivers, and providers), the ACHIEVE research team will determine what core components of successful transitional care programs are most effective and what unique features are useful for particular populations under what specific circumstances. Given that communities are constantly changing, our proposed study also will evaluate contextual factors attempting to pinpoint influences of specific characteristics or changes that occurred in the community on trends in patient and caregiver centered outcomes.

Implementation research focuses on understanding how programs are implemented, translated, replicated, and disseminated in “real-world” settings and can consider any aspect of implementation, including the factors affecting implementation, the processes and the results of implementation, how to introduce potential solutions into a health system or how to promote their large-scale use and sustainability. It expands the focus of traditional research from discovering what works to also discovering how the implementation works in specific contexts, and emphasizes establishing external validity so that knowledge about how to effectively implement programs can be applied to a wide range of settings [31]. Undertaking a comprehensive analysis of implementation is particularly important when studying the TC programs because the models are complex, includes multiple interacting components, and is intended to be adapted to fit the needs of organization setting and patient population. Additionally, the healthcare and community organizations in which the TC program is being introduced are complex adaptive systems -- dynamic settings that co-evolve with the TC program as it is implemented.

The Project ACHIEVE research team possesses vast experience implementing care transition interventions [1, 2, 5, 6, 32], and has previously published models for implementation [33]. Leveraging this experience and expertise, we will address organizational and contextual dynamics that affect decisions to adopt evidence-based programs, and feasibility of implementation with fidelity to the original model when new users adopt established programs. Importantly, we also will use new knowledge gained from querying patients and caregivers to address their acceptance of care transition interventions. Finally, partnerships with key HENs (HRET, AEH, JRS), the National Association for Area Agencies on Aging, and patient and caregiver advocacy groups will enable us to reach a broad population and increase uptake.

## Abbreviations

ACHIEVE: achieving patient center care and optimized healthcare transitions by evaluating the value of evidence
TC: transitional care
TCC: transitional care component
CMS: center for medicare and medicaid services
SNF: skilled nursing facility
PCORI: patient centered outcome research institute
QIO ICPC: quality improvement organizations’ integrating care for populations and communities
CCTP: community-based care transitions program
HEN: hospital engagement network
PRAiHS: promoting action on research implementation in health services
ISF: interactive systems framework
CFIR: consolidated framework for implementation research
RE-AIM: reach, effectiveness, adoption, implementation, maintenance
CBPR: community-based participatory research
SAG: stakeholder advisory group
FF: fractional factorial
GLLAMM: general linear latent and mixed models
CATI: computer-assisted telephone interviewing
GEE: generalized estimating equation
GLMM: general linear mixed model
