# Erratum to: Project ACHIEVE – using implementation research to guide the evaluation of transitional care effectiveness

**DOI:** 10.1186/s12913-016-1560-x

**Published:** 2016-08-01

**Authors:** Jing Li, Jane Brock, Brian Jack, Brian Mittman, Mary Naylor, Joann Sorra, Glen Mays, Mark V. Williams, for the Project ACHIEVE Team

**Affiliations:** 1 Administrative Director of the Center for Health Services Research, Assistant Professor of Internal Medicine, University of Kentucky, Lexington, KY USA; 2 Care Transitions Theme Support Center, Telligen, Englewood, CO USA; 3 Family Medicine, Boston University School of Medicine, Boston, MA USA; 4 Research Scientist, Research and Evaluation, Kaiser Permanente, Pasadena, CA USA; 5 US Department of Veterans Affairs Greater Los Angeles Healthcare System, VA Center for Implementation Practice and Research, Los Angeles, CA USA; 6 UCLA School of Medicine, UCLA Clinical Translational Science Institute, Los Angeles, CA USA; 7 Director of NewCourtland Center for Transitions and Health, University of Pennsylvania School of Nursing, Philadelphia, PA USA; 8 Westat, Washington D.C., USA; 9 National Coordinating Center for Public Health Services & Systems Research, University of Kentucky, Lexington, KY USA; 10 Center for Health Services Research, Department of Internal Medicine, University of Kentucky, Kentucky Clinic J525, Lexington, KY 40536-0284 USA

## Erratum

After publication of the original article [1] it was brought to our attention that the following declaration should be included:

‘The statements presented in this publication are solely the responsibility of the authors and do not necessarily represent the views of the Patient-Centered Outcomes Research Institute (PCORI), its Board of Governors or Methodology Committee’.

Also, author Joann Sorra was incorrectly included as Joanna Sorra. The correct spelling of Joann Sorra’s name is included in the author list of this erratum.
